# Strengthening Policy Coherence to Link Healthy Hydration with Water Security in the United States^☆^

**DOI:** 10.1016/j.advnut.2026.100696

**Published:** 2026-07-06

**Authors:** Vivica I Kraak, Eric Otoo-Annan, Luke Juran

**Affiliations:** 1 Department of Human Nutrition, Foods, and Exercise, Wallace Hall, Virginia Polytechnic Institute and State, University (Virginia Tech), Blacksburg, VA, United States; 2 Department of Human Nutrition, Foods, and Exercise, Wallace Hall, Virginia Tech, Blacksburg, VA, United States; 3 Department of Geography and the Virginia Water Resources Research Center, Wallace Hall, Virginia Tech, Blacksburg, VA, United States

**Keywords:** water security, healthy hydration, functional beverages, bottled water, regulation, policy coherence

## Abstract

Water security ensures the availability, accessibility, and acceptability of safe water for drinking, hygiene, and other purposes for individuals and communities to meet daily and future needs. An estimated 2.2 million Americans experience water insecurity linked to poor diet quality and adverse health outcomes. This perspective article summarizes the scientific evidence about water security and healthy hydration to understand the opportunities and challenges for government agencies to strengthen the links and policy coherence across both areas for the population. We focus on the United States context because of the complex policy landscape of government agencies with different regulatory oversight for promoting healthy hydration and water security, and a weakly regulated yet growing multibillion-dollar bottled water, functional beverage, and hydration supplement industry marketed to Americans. First, we describe healthy hydration recommendations and United States fluid intake trends across the lifespan. Second, we examine the evolving industry that produces and markets hydration products, including bottled water, soft drinks, nonalcoholic functional beverages, and dietary supplements. Third, we discuss the United States government agencies that have regulatory oversight to protect and educate the public to ensure the safety of bottled water and publicly supplied drinking water and enforce policies requiring that businesses use truthful advertising and marketing claims for functional beverages and supplements. We offer recommendations for United States government agencies and other stakeholders to strengthen policy coherence and improve coordination of guidelines and legislation to ensure affordable, healthy hydration and water security for all Americans.

## Graphical abstract

**Figure:**
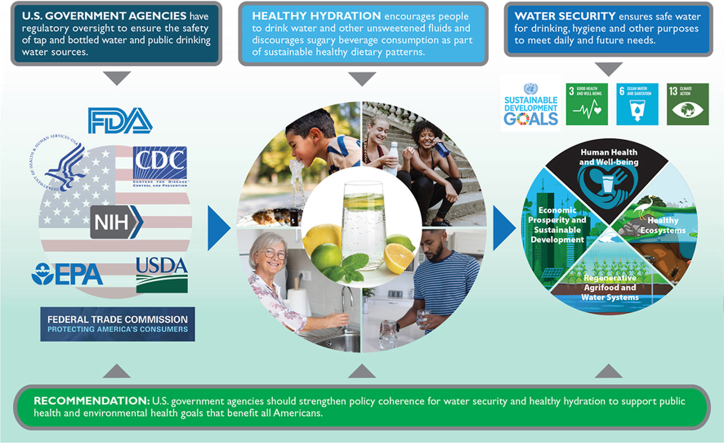

**Statement of significance**

This perspective article synthesizes the scientific evidence about water security and healthy hydration and the evolving multibillion-dollar bottled water, functional beverage, and hydration product industry in the United States. We examined the opportunities and challenges for government agencies and other stakeholders to strengthen policy coherence and improve coordination of guidelines and legislation to ensure affordable, healthy hydration and water security for all Americans.

## Introduction

**FIGURE 1:**
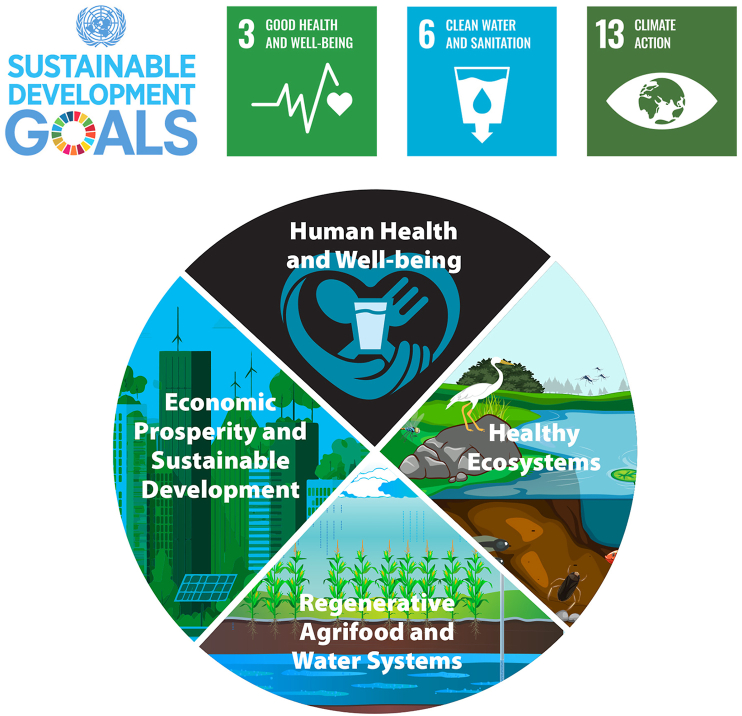
Water security for people and the planet. An original figure created by QDesign and adapted from references [[5], [6], [7], [8]] with permission.

In 2010, the United Nations (UN) declared that safely managed drinking water is a basic human right and essential for realizing all human rights [1]. However, between 2.1 and 4.4 billion people worldwide lack access to safe and clean water due to complex challenges, including policy, infrastructure, poverty, and societal inequities [[2], [3], [4]]. Threats to the UN-established universal right to safe water have become even more urgent as the impacts of climate change affect both the distribution of water resources and water use in dynamic ways [5]. Figure 1 [[5], [6], [7], [8]] shows that water security is important for people and the planet and is linked to many UN Sustainable Development Goals (SDGs), especially SDG 3 (good health and well-being), SDG 6 (clean water and sanitation), and SDG 13 (climate action). Water security ensures the availability, acceptability, and accessibility of safe water for drinking, hygiene, and other purposes to meet daily and future needs of individuals and communities [6,9]. Figure 1 shows that water security supports human health and well-being, healthy ecosystems, economic prosperity for livelihoods and sustainable development, and regenerative agrifood and water systems under a changing climate [6,9,10].

Advancing the water security targets for the UN SDG 6 promotes health and well-being (SDG 3) by protecting communities from water contamination and waterborne diseases and supports SDG 13 (climate action) targets for climate change mitigation, adaptation, and resilience [[6], [7], [8], [9], [10], [11]]. Mitigation involves reducing emissions that cause climate change, which affect rainfall and water distribution; adaptation involves managing climate change risk (e.g., water conservation); and resilience represents actions to address climate change (e.g., sustainable water management) [7,10,11].

Drinking adequate amounts of water daily is essential for healthy skin, cognitive, organ, and gut health, to compensate for sweating while working in hot temperatures, and for physical and mental well-being [12,13]. Healthy hydration encourages people to consume water and unsweetened fluids and discourages sugary beverages. Ensuring that individuals and communities have consistent access to safe drinking water at home and in public settings supports both water security and healthy hydration for Americans [12].

However, ∼2.2 million Americans live in homes without running water or adequate plumbing, and millions more do not trust the safety of their tap or well water [14,15]. Water insecurity is an environmental injustice, especially for ethnically and racially diverse Americans living in poor urban and rural settings who do not like the taste and odor of their public drinking water and view bottled water as more convenient [12,[14], [15], [16], [17]]. Water insecurity in the United States is a structural barrier to healthy hydration associated with the increased intake of expensive bottled water or sugary beverages instead of tap water, which may contribute to poor diet quality and adverse health outcomes [[18], [19], [20], [21], [22]].

A striking paradox of the water insecurity experienced by low-income Americans is the multibillion-dollar beverage industry that markets branded bottled water, sugary beverages, and artificially sweetened beverages to the United States population. The Coca-Cola Company, PepsiCo, Nestlé, and Danone are the leading United States bottled water and sugary beverage firms that contribute to substantial public health, social, and ecological costs, which adversely impact individuals, populations, and communities. Health concerns are linked to an increased risk of chronic diseases, including obesity, type 2 diabetes, heart disease, and cancer. Although these beverage firms generate jobs and invest in communities through corporate philanthropy, the social costs are the privatization of community water resources. The ecological costs include the excessive production of single-use plastic pollution and waste (e.g., bisphenol A, phthalates, and microplastics) with low recycling rates [[23], [24], [25]]. The United States also has an expanding market for nonalcoholic functional beverages that contain bioactive ingredients, which are marketed to the population with limited evidence of health benefits [[26], [27], [28]].

This perspective article synthesizes the scientific evidence about water security and healthy hydration to understand the opportunities and challenges for government agencies to strengthen the links and policy coherence across both areas for the population. We focus on the United States context because of the complex policy landscape of government agencies with different regulatory oversight for promoting healthy hydration and water security, and a weakly regulated yet growing multibillion-dollar industry that produces and markets bottled water, functional beverages, and dietary supplements for healthy hydration and wellness to Americans. First, we examine recommendations issued by scientific advisory bodies and organizations for water and fluid needs to support healthy hydration across the lifespan. Second, we synthesize the literature on the United States market growth and the firms that manufacture and market branded bottled water and nonalcoholic, ready-to-drink energy and sports drinks, functional beverages, and hydration supplements. Third, we examine United States federal government agencies that regulate the safety and health benefits of tap and bottled water, functional beverages, and hydration supplements. Fourth, we suggest actions for government agencies and other stakeholders to strengthen the links and policy coherence to ensure affordable, healthy hydration and water security for all Americans.

## Healthy Hydration Recommendations and United States Fluid Intake Trends across the Lifespan

Healthy hydration encourages people across the lifespan to select water and nutrient-dense fluids to support essential biological and physiological functions in the human body, such as optimizing blood flow for cardiovascular health, transporting nutrients, regulating body temperature, and removing waste [29]. Healthy hydration promotes fluid amounts according to a person’s specific needs, preferences, and requirements and discourages the intake of beverages that contain excessive added sugars [29]. Although fluid needs differ based on age, sex, and life stage, expert bodies recommend that adult men consume ∼15.5 cups/8-ounce (3.7 L) daily, and adult women consume ∼11.5 cups/8-ounce (2.7 L) daily [29].

FAO and WHO recommend that governments encourage water as the default beverage to support sustainable healthy diets [30]. In 2005, the United States National Academy of Medicine recommended adequate intake amounts for individuals across the lifespan in the Dietary Reference Intakes (DRIs) to support optimal health [31]. The Dietary Guidelines for Americans (DGA) 2020–2025 are based on the DRIs, which recommend that people choose water and nutrient-rich nonalcoholic beverages such as coffee, tea, and flavored waters without added sugars or fats and to replace sugary beverages with water or unsweetened beverages, but do not issue explicit recommendations for artificially sweetened or functional beverages [32]. The American Heart Association used the DGA recommendations to encourage healthy hydration as part of a daily routine and promoted water and water-rich foods (i.e., fruits and vegetables) [33]. The American Heart Association discouraged sugary fruit juices, sodas, and sports drinks and advised adult women, children, and teenagers to consume no >6 teaspoons (25 g) of added sugars daily and adult men to consume no >9 teaspoons (36 g) daily [33].

The 2025 Dietary Guidelines Advisory Committee (DGAC) scientific report recommended that the 10th edition of the DGA 2025–2030 should emphasize plain drinking water as the primary beverage for hydration, followed by nutrient-dense beverages including fat-free and low-fat milk, fortified soy beverages, and 100% juice [34]. The report also recommended that people reduce sugary beverages that contain calories but limited nutrients, but did not address the health benefits of nonsugar-sweetened (NSS) beverages with artificial or natural sweeteners or functional beverages [34].

The DGA 2025–2030 report released by the Secretaries of the Department of Health and Human Services and USDA encouraged Americans to choose still or sparkling water, unsweetened beverages, and full-fat dairy with no added sugars [35]. The DGA 2025–2030 report also advised that Americans limit beverages that include artificial flavors, dyes, preservatives, and low-calorie NSS or sugar substitutes [35]. The graphic inverted pyramid that replaced MyPlate featured full-fat whole milk but showed no visual reference to encourage water or unsweetened beverages or discourage NSS or artificially sweetened beverages [35].

**FIGURE 2:**
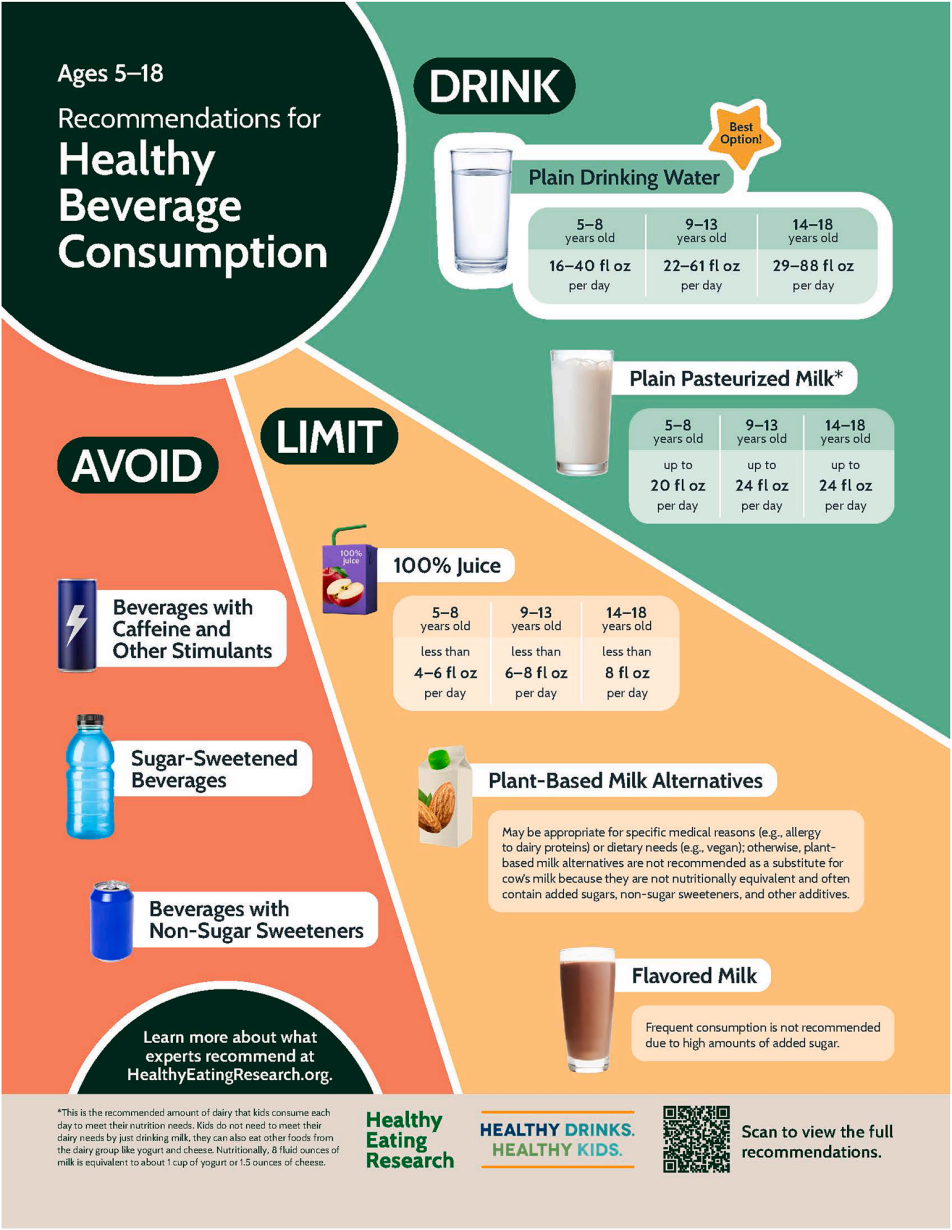
Healthy beverage recommendations for United States children and teens, aged 5 to 18 y. Reproduced with permission from Healthy Eating Research.

Figure 2 shows the healthy beverage recommendations for children and teens aged 5 to 18 y published in 2025 by several United States organizations [36]. The recommendations encourage plain water and unsweetened pasteurized milk as primary beverage choices. They also suggest that children limit their intake of 100% juice, plant-based milk alternatives, and flavored milks and avoid drinking beverages that contain sugar, artificial sweeteners, and caffeine [36].

### United States fluid intake trends across the lifespan

NHANES data (2001–2019) show that United States adults who followed healthy beverage patterns comprising water, low-fat milk, and unsweetened coffee and tea had a lower risk of cardiovascular disease and all-cause mortality than adults with unhealthy beverage patterns with fruit juices, artificially sweetened beverages, sugary beverages, whole-fat milk, and alcohol [37].

In the United States diet, carbonated soft drinks, fruit drinks, flavored milk, and coffee and tea contribute to fluids but provide excessive added sugar intake, resulting in lower diet quality for adults, teens, and children [38]. Between 2016 and 2018, the average United States adult woman and man consumed ∼10 cups and 11 to 12 cups (equivalent to 8 ounces/240 mL per cup) of fluid daily, respectively, which is below the National Academy of Medicine’s DRI target of 11.5 to 15.5 cups of fluid daily [39,40]. The DRI fluid target for children is 4 to 8 cups daily. In 2017–2018, United States children and teens aged 2 to 19 y consumed 5.5 cups of fluid daily; half was water and one-third was sweetened beverages.

Between 2011 and 2018, the primary sources of added sugars associated with poorer nutrient adequacy and diet quality were soft drinks and sweetened teas for adults and fruit drinks, sports, or energy drinks for children and teens [41,42]. The National Survey of Children’s Health (2021–2023) data found that sugary beverages are the main source of added sugars in the diets of young children, and an estimated 1 in 3 children aged 1 to 5 y consumes a sugary beverage weekly [43].

Coffee, tea, soft drinks, and energy drinks are the main sources of caffeine consumed by United States adults and children [44]. The 2025 DGAC report recommends that ≤400 mg daily of caffeine is safe for adults but does not provide a specific recommendation for children and teens [35,37]. The average caffeine intake is 210 ± 1.5 mg, with the highest intake by United States adults aged 50 to 64 y (246 ± 4.5 mg/day) and the lowest intake by children aged 2 to 5 y (42 ± 2.4 mg/day) [44].

### The United States industry marketing of hydration, health, and wellness

The International Bottled Water Association classifies bottled or packaged water marketed to consumers into various categories, including spring, purified, mineral, sparkling, artesian, and well water [45]. Most Americans obtain their water from bottled water, municipal water, and filtered water sources at home or restaurants [45]. The Coca-Cola Company, PepsiCo, Inc, and Nestlé dominate the United States bottled water, carbonated soft drinks, and functional beverage market [25,26,46]. In 2024, the United States bottled water market was approximately USD $112 billion, and each of the 4 major bottled water brands (i.e., Aquafina, Dasani, Glacéau, and Poland Spring) generated approximately USD $1 billion for bottled water [47].

Beverages that provide sweeteners and energy (>50 calories per 8 fluid ounces) but limited nutrients or beneficial ingredients are not considered functional beverages. However, sugary beverage manufacturers are innovating with new flavors and ingredients for a growing functional beverage market. Nonalcoholic functional beverages provide benefits beyond basic hydration for human health and wellness to enhance physical and/or immune function, gut health, or cognitive performance. Functional beverages may contain 1 or more bioactive ingredients, including micronutrients (e.g., vitamins and minerals); electrolytes (i.e., magnesium, potassium, and sodium); energy boosters (e.g., caffeine, guarana, and taurine); and health-enhancing substances (e.g., herbs, adaptogens, creatine, lactoferrin, probiotics, prebiotics, polyphenols, and cannabidiol) [[48], [49], [50], [51]]. Hydration supplements may be powders or tablets reconstituted with water [52].

**FIGURE 3:**
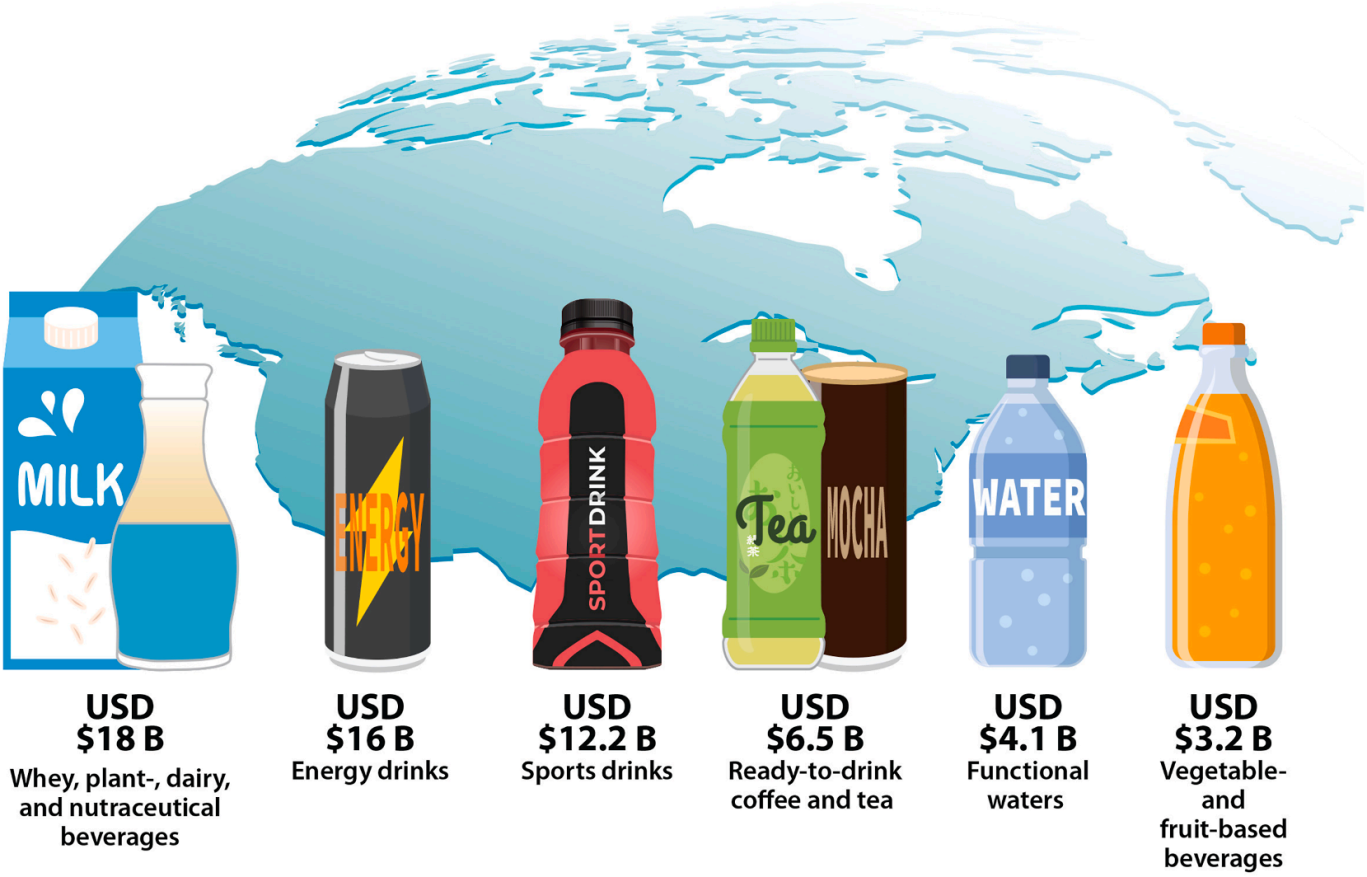
Marketing landscape for functional beverages in North America, 2024. Adapted from references [45,48,50,55,56] with permission.

Marketing research suggests that the global functional beverage market was valued at >$150 billion by 2024 and may reach $250 billion by 2030 [52,53]. Figure 3 shows that the North American functional beverage market was estimated at USD $60 billion in 2025 and may reach USD $80 billion by 2030 [45,48,50,[54], [55], [56]].

The functional beverage market categories include the following: *1*) whey, plant, dairy, and nutraceutical beverages; *2*) energy drinks, shots, and mixers; *3*) sports drinks; *4*) ready-to-drink coffee and tea; *5*) unflavored and flavored functional waters; and *6*) vegetable- and fruit-based beverages. In 2024, energy drinks (e.g., Red Bull, Monster, Rockstar, Bang, 5-hour Energy, and NOS) generated USD $16 billion, sports drinks (e.g., Bodyarmor, Powerade, Gatorade, Prime, and Electrolit) generated USD $12.2 billion, and functional water brands such as Propel Fitness Water (PepsiCo) and Smartwater (The Coca-Cola Company) generated >USD $4.1 billion [[48], [49], [50],^55^,^56^].

Beverage firms use advertising and integrated marketing communications strategies to promote functional beverages to Generation Alpha children and adolescents, Generation Z adults, and athletes who consume these products for hydration, weight management, gut health, and to enhance physical performance, health, and wellness [[57], [58], [59], [60]].

Sports and energy drink products have different formulations that may provide benefits to or adversely impact one’s diet, dental and physical health, and cognitive well-being [[60], [61], [62], [63], [64]]. Antonio et al. [61] reviewed the benefits and misconceptions of energy drinks and concluded that caffeine was the single most important ingredient for users in this beverage category, which is associated with improved alertness, concentration, memory, and attention. However, poorly timed consumption may disrupt sleep patterns and impair cognitive activities, and some formulations may cause acute adverse cardiovascular effects. Furthermore, energy drinks provide only modest benefits for long-term endurance sports activities [61].

Orrù et al. [63] reviewed evidence on the influence of macronutrient-enriched functional sports beverages (including sports drinks) on the health, performance, and recovery of athletes and concluded that there is no optimal formulation due to individual variation. Muñoz-Urtubia et al. [64] recommend water as the primary beverage for nonathletes and that sports drinks should have warning labels to inform consumers about the potential harms to nonathlete consumers. Sports nutrition scientists often receive funding from beverage companies to develop guidelines for sports beverages through industry-sponsored research and sports medicine organizations [65]. Although marketed to the public, sports drinks are developed for elite athletes; registered dietitian nutritionists and exercise scientists recommend water as the primary beverage choice for nonathlete consumers [64,65].

## Regulatory Oversight for Beverages, Supplements, and Health

Several United States federal government agencies collaborate to educate the public or enforce regulations, monitor and/or conduct research on the safety and healthfulness of water, functional beverages, and hydration supplements. These agencies include the CDC, Environmental Protection Agency (EPA), FDA, Federal Trade Commission (FTC), National Institute of Environmental and Health Sciences, and USDA. The CDC provides educational resources for the public that encourage drinking safe water and healthy, nutrient-dense, unsweetened beverages instead of sugary beverages to optimize human health [66]. The CDC has developed guidelines for Americans to treat water and reduce zoonotic disease transmission from bacteria and parasites while hiking, camping, and traveling [67].

The EPA regulates environmental water quality based on the provisions in the Clean Water Act of 1972, which aims to support water security by regulating pollutant discharges into the United States water sources [68]. The EPA’s National Pollutant Discharge Elimination System is a permitting program that addresses water pollution by setting water quality standards and regulating point sources that discharge pollutants to United States waters [69]. Evaluation of the Clean Water Act and other voluntary efforts to control or reduce pollution from agriculture and urban runoff has shown that the benefit-cost ratio of policies has had minimal positive impacts [70].

Tap water quality is regulated by the 1974 Safe Drinking Water Act and amendments of 1986 and 1996, which established standards for >90 contaminants in drinking water. These contaminants include naturally occurring minerals (e.g., fluoride and sodium); geogenic and human-induced chemicals such as arsenic, lead, and copper; microbial contaminants (e.g., *Escherichia coli*) and physical properties (e.g., turbidity) [71]. The Safe Drinking Water Act also regulates “forever chemicals” called per- and polyfluoroalkyl substances in drinking water, specifically perfluorooctanoic acid and perfluorooctane sulfonic acid, which are synthetic chemicals used to make products resistant to water and grease [72]. The EPA maintains an inventory of chemical substances required by the Toxic Substances Control Act of 1979, which has grown over decades, yet the EPA regulates only a small proportion of chemical contaminants in the United States water sources [73]. In April 2026, the Health and Human Services and EPA heads announced plans to monitor microplastics and pharmaceutical compounds in drinking water and study their risks to human health to inform new regulatory policies. They also proposed adding microplastics and pharmaceuticals to the EPA’s Contaminant Candidate List but took no other regulatory actions to reduce toxic chemicals in drinking water provided to millions of Americans [73].

In many urban United States settings, high concentrations of lead, a neurotoxin, were found in the tap water of private homes, schools, and childcare centers and attributed to an aging water infrastructure [74]. An estimated 158 million Americans are exposed to forever chemicals in their drinking water, which have been linked to many adverse health outcomes [75]. The health burden related to forever chemicals, phthalates, bisphenols, and pesticides in food and water supplies is estimated at USD $2.2 trillion annually [76]. The National Institute of Environmental and Health Sciences conducts research on the potential health effects of these contaminants and how to protect the United States public from unsafe water [77].

In 2024, the EPA announced new drinking water regulations for state and local governments to test water for 6 toxic forever chemicals, including perfluorooctanoic acid and perfluorooctane sulfonic acid, that affect >100 million Americans [78]. The Trump Administration’s EPA Administrator announced the largest government deregulatory action agenda in history in March 2025 to remove legal protections for the American public, environment, and water supply while undermining the United States government’s commitments to climate action [79].

The maximum contaminant level for fluoride is 4.0 mg/L (enforceable), whereas the secondary safety standard (nonenforceable) is 2.0 mg/L to prevent mild or moderate dental fluorosis and dental cavities among children [80]. The CDC uses a Tracking Network to monitor contaminants in drinking water that have public health significance [81], such as monitoring municipal and state water supplies through the Water Fluoridation Reporting System [81].

In 2022, an estimated 72% of United States municipal water systems were fluoridated, with state and local governments determining the concentration [82]. Despite strong scientific evidence demonstrating that water fluoridation is safe and effective for preventing dental decay [82], some state and local governments have passed legislation to remove fluoride from public water based on unsubstantiated health concerns. In 2025, 12 states had laws that mandated fluoridation, whereas Utah and Florida enacted laws that prohibited public water fluoridation because of concerns about adverse health outcomes [83]. In January 2026, the EPA announced that it would conduct an expedited review to determine safe concentrations of fluoride in drinking water to address unsubstantiated concerns raised by the Make America Healthy Again movement that fluoride in municipal water has adverse effects on human health [84]. These legislative actions conflict with the longstanding position of the CDC [82] and American Dental Association, which stated that the removal of fluoride from community water systems will undermine dental health, representing an estimated cost of USD $46 billion (attributed to an increase in health care and other indirect costs) over 5 y [85].

The FDA sets good manufacturing practices for processing bottled water, which it defines as mineral, sparkling, spring, and purified water. The FDA has regulatory responsibility for the safety and labeling of ingredients in bottled water and functional beverages that are required on the Nutrition Facts panel [82]. The International Bottled Water Association represents United States and international bottlers, distributors, and suppliers whose members work with the FDA to ensure that bottled water meets United States quality standards and adheres to a voluntary bottled water code of practice [45]. The FDA’s health standards for bottled water differ from the EPA’s public water safety requirements, and contaminants have been found in bottled water that may have adverse effects on human health [84].

The FDA regulates bottled water, carbonated soft drinks, and functional beverages (i.e., sports and energy drinks) as conventional packaged food products [86]. Certain products may be regulated as dietary supplements depending on the ingredients and labeling requirements. The 2025 DGAC [34] and FDA [86] recommend that ≤400 mg of caffeine daily (approximately 2–3 12-ounce cups of brewed coffee) is safe for adults to consume without adverse effects, but no specific caffeine guidelines have been issued for children and teens despite expert recommendations that young people avoid caffeine (Figure 2) [36].

The FDA’s Adverse Event Reporting System database tracks medical events and consumer complaints for caffeine-containing products and side effects related to using dietary supplements [87]. The FDA has the authority to remove a functional beverage or hydration supplement from the United States market through the Dietary Supplement Health and Education Act of 1994, but the agency must prove that a product has adverse health effects or that it does not contain what is listed on the label [88].

Companies are responsible for ensuring the regulatory status and safety of the ingredients in their beverage products before marketing. The FDA provides a premarket regulatory program that allows certain ingredients to be used as additives if they are deemed generally recognized as safe [89]. The FDA has approved 6 high-intensity artificial sweeteners including acesulfame potassium, advantame, aspartame, neotame, saccharin, and sucralose and 3 plant fruit-based sweeteners (e.g., monk fruit, stevia, and thaumatin) as safe additives that may be used by the food and beverage industry for products marketed in the United States if consumed within established acceptable daily intake amounts [89]. There is currently a lack of policy coherence between the FDA’s approval of 9 artificial or natural low- or no-calorie sweeteners and the 10th DGA 2025–2030 recommendation that individuals avoid consuming products with additives such as artificial NSS [35]. Because of evolving evidence about the potential harms of NSS beverages on diet and health, a range of public policies have been proposed or enacted at local, state, and federal levels to establish standards, restrict or ban NSS in food and beverage products [90].

The FDA and FTC coordinate the regulation and enforcement of marketing guidelines for claims made by manufacturers and social media influencers [59] who promote functional beverages and supplements to enhance hydration, health, and wellness. In 2022, the FTC issued updated guidelines for businesses to provide scientific evidence of health-related claims on products to ensure truthful information that is not misleading or deceptive to the average consumer [91]. The FTC requires health benefit claims to be substantiated by randomized controlled human clinical trials, and advertisers must disclose any significant limitations for a claimed benefit [89]. In 2023, the FTC updated guidelines for endorsement and social media influencers to disclose payment or sponsorship for specific products [90], including bottled functional and artificially sweetened beverages and dietary supplements.

The EPA and FTC share regulatory oversight for developing and enforcing ecolabeling and environmental or sustainability marketing claim guidelines [92,93]. A 2024 lawsuit was filed in Los Angeles County, California, against The Coca-Cola Company and PepsiCo for misleading consumers about plastic beverage containers presented as recyclable without disclosing their environmental impacts. Research suggests that most single-use plastic containers end up in landfills or terrestrial and aquatic ecosystems and become microplastics, but these claims were dismissed by the California state court [94].

The USDA administers federal food and nutrition programs for low-income Americans. Although the Thrifty Food Plan encourages drinking tap water and the electronic benefits provided through the Supplemental Nutrition Assistance Program (SNAP) may be used by participants to purchase bottled and bulk water at authorized retailers, water filters may not be purchased by eligible recipients [95]. By June 2026, 23 states had approved SNAP restriction waivers to discourage sugary beverages, including energy drinks and fruit and vegetable drinks with added sugars [95]. The USDA’s Food and Nutrition Administration could offer education for healthy hydration, given that funding for the SNAP-Education was discontinued across all states after the One Big Beautiful Bill law was enacted in July 2025.

The USDA’s nutrition standards for school meals and smart snacks allowed plain water with or without carbonation, unflavored or flavored fat-free milk or fortified soy milk, and 100% fruit or vegetable juice with no added sweeteners to be served to children [96]. The United States schools are encouraged to make plain water available for free in locations where students are served meals, and water can be provided through fountains, pitchers, cups, or faucets where students can serve themselves. The National Drinking Water Alliance is a network of United States organizations that collaborate to ensure that children can access safe drinking water where they live, learn, and play and can play an important role in healthy hydration and water security in communities [97].

In January 2026, President Trump signed the Whole Milk for Healthy Kids Act that enabled schools to serve whole and 2% milkfat cow's milk to children in the National School Lunch Program but not the School Breakfast Program [98]. This law aligned full-fat dairy as part of a healthy dietary pattern in the DGA 2025–2030 and excluded fluid milk from the saturated fat content calculations for schools [98]. The legislation allows flavored and unflavored organic or nonorganic whole, reduced-fat, low-fat, and fat-free fluid milk and lactose-free fluid milk; although parents and legal guardians may submit a written statement to request milk substitutions, such as fortified soy milk, for their children [98]. Ongoing policy enforcement and monitoring are essential to ensure that water and other nutrient-dense beverages served to students align with the USDA’s standards and school wellness policies and to evaluate this change to assess the influence on children’s diet quality and health. Improving restaurant policies and practices may also encourage water as the healthy default beverage for children and teens. A 2026 study compared policies across 4 states and 64 unique jurisdictions but found inconsistencies with the implementation and enforcement of the healthy default beverage policies [99].

## Strengthening Policy Coherence for the United States Healthy Hydration and Water Security

The 2025 UN SDG Report showed that the United States ranked 44 out of 193 member states for implementing policies and actions to achieve the SDG Agenda targets [100]. Compared with other high-income countries, the United States has not made measurable progress in implementing SDG 6 (clean water and sanitation) and SDG 13 (climate action) by 2030, which are important for achieving water security and healthy hydration [100]. Water security is an important component of the One Health approach to balance and optimize the health of people, animals, ecosystems, and the planet [101]. Pepin et al. [101] recommend strategies for countries to since One Health approaches into SDG monitoring, which will require governments to implement cross-sectoral policies and behavioral change strategies.

Several challenges remain for United States government agencies to strengthen policy coherence to promote healthy hydration and water security for all Americans. The federal government has reduced funding to <5% of the total investment in water and wastewater infrastructure nationally, which increases the responsibility of clean water compliance and strengthening infrastructure investment to state and local authorities and customers who pay for public water [102]. Communities may not trust the water quality when public notices raise concerns about their tap water, forcing residents to use alternative sources (e.g., bottled water) when their local drinking water is unsafe. Yet, bottled water regulations are weaker than those for tap water to ensure safety and quality. Major beverage firms have secured the legal rights to use aquifers in communities, supported by state and local permits or leases, which redirect water resources and create water shortages for residents near bottling centers [103]. Digital tech firms are also building artificial intelligence (AI) data centers across the United States in states that require large amounts of water to generate electricity, which have hidden environmental costs that may increase water insecurity [104].

Strengthening United States governance for healthy hydration and water security requires policy coherence to reinforce, incentivize, and hold stakeholders accountable for actions across sectors and settings to achieve personal, ecological, and planetary health goals [103]. For example, a more purposeful integration of policies that support the water–food–energy nexus in which decisions for activities such as irrigation, agrochemical use, **AI**, data center siting, and water abstractions are needed to balance competing human and ecological needs. This not only requires holistic policymaking, prudent spending, and regulatory oversight but also a robust and fair judicial system that holds industries and private-sector actors accountable for their impact on communities and the environment.

Policy coherence reinforces government actions across departments and agencies to maximize opportunities, expose trade-offs, minimize inconsistencies, and support public health goals and facilitates both water security and healthy hydration for the United States population to access safe and affordable water and affordable healthy beverages [105]. Policy inventories at local, tribal, state, and national levels can inform the United States government, business, and civil society about how to promote water security and healthy hydration within and across different settings [106]. Public health professionals, nutritionists, and civil society organizations can make impactful changes by providing clean water dispensers in schools and expanding free water availability and access in communities.

The EPA requires community water systems to issue an annual Consumer Confidence Report to residents that describes water quality based on data from the previous year [107]. Most states in the United States offer public education to raise awareness about the need for annual testing of water quality and data about well water. However, these states lack legislation that requires testing, landlord and property disclosures, and notices of contamination for privately owned wells for water [108]. Increased advocacy is needed to strengthen policy coherence and secure adequate funding to improve federal, state, and local policy coordination and legislation for implementing, monitoring, and evaluating laws, voluntary guidelines, and programs that protect water security and healthy hydration for Americans [[103], [104], [105], [106], [107], [108]].

In conclusion, a coherent policy response is urgently needed to address the drivers of water insecurity experienced by 2.2 million Americans while encouraging affordable, healthy hydration. Healthy hydration encourages the consumption of plain water, nutrient-dense beverages, naturally sweetened water, and whole fruits and vegetables for beneficial nutrients instead of buying expensive functional beverages. Healthy hydration also limits beverages with added sugars, artificial sweeteners, and excessive caffeine. Beverages that provide sweeteners and energy but limited nutrients or beneficial ingredients are not considered functional beverages. Functional beverages provide benefits beyond basic hydration for human health and wellness to enhance physical and/or cognitive performance or immune function. Sugary beverage manufacturers are innovating to support a growing functional beverage market worth USD $60 billion in 2024 in North America. Stronger government leadership, urged by civil society and the public, will help to implement guidelines, policies, and laws at the federal and state levels. Diverse stakeholders will need to coordinate actions across agencies and sectors to improve the potable water infrastructure and regulate a growing functional beverage market to ensure affordable, healthy hydration and water security for all Americans.

## Author contributions

The authors’ responsibilities were as follows – VIK: Conceptualization, writing—original draft and review and editing. EOA: Conceptualization, writing—review and editing. LJ: Conceptualization, writing—review and editing, and all authors: read and approved the final manuscript.

## Declaration of Generative AI and AI-assisted technologies in the writing process

The authors declare that no generative AI or AI-assisted technologies were used in the writing of this manuscript.

## Funding

VIK received funding from the United States Department of Agriculture (USDA) National Institute of Food and Agriculture Hatch Project VA-160189.

## Conflict of interest

All authors report no conflicts of interest related to the content of this paper.
